# Regulation of *ddb2* expression in blind cavefish and zebrafish reveals plasticity in the control of sunlight-induced DNA damage repair

**DOI:** 10.1371/journal.pgen.1009356

**Published:** 2021-02-05

**Authors:** Haiyu Zhao, Hongxiang Li, Juan Du, Giuseppe Di Mauro, Sebastian Lungu-Mitea, Nathalie Geyer, Daniela Vallone, Cristiano Bertolucci, Nicholas S. Foulkes

**Affiliations:** 1 Institute of Biological and Chemical Systems, Karlsruhe Institute of Technology, Eggenstein-Leopoldshafen, Germany; 2 School of Life Sciences, Lanzhou University, Lanzhou, PR China; 3 State Key Laboratory of Freshwater Ecology and Biotechnology, Institute of Hydrobiology, Chinese Academy of Sciences, Wuhan, PR China; 4 Department of Life Science and Biotechnology, University of Ferrara, Ferrara, Italy; 5 Department of Biomedical Sciences and Veterinary Public Health, Swedish University of Agricultural Sciences, Uppsala, Sweden; National Institute of Environmental Health Sciences, UNITED STATES

## Abstract

We have gained considerable insight into the mechanisms which recognize and repair DNA damage, but how they adapt to extreme environmental challenges remains poorly understood. Cavefish have proven to be fascinating models for exploring the evolution of DNA repair in the complete absence of UV-induced DNA damage and light. We have previously revealed that the Somalian cavefish *Phreatichthys andruzzii*, lacks photoreactivation repair via the loss of light, UV and ROS-induced *photolyase* gene transcription mediated by D-box enhancer elements. Here, we explore whether other systems repairing UV-induced DNA damage have been similarly affected in this cavefish model. By performing a comparative study using *P*. *andruzzii* and the surface-dwelling zebrafish, we provide evidence for a conservation of sunlight-regulated Nucleotide Excision Repair (NER). Specifically, the expression of the *ddb2* gene which encodes a key NER recognition factor is robustly induced following exposure to light, UV and oxidative stress in both species. As in the case of the *photolyase* genes, D-boxes in the *ddb2* promoter are sufficient to induce transcription in zebrafish. Interestingly, despite the loss of D-box-regulated photolyase gene expression in *P*. *andruzzii*, the D-box is required for *ddb2* induction by visible light and oxidative stress in cavefish. However, in the cavefish *ddb2* gene this D-box-mediated induction requires cooperation with an adjacent, highly conserved E2F element. Furthermore, while in zebrafish UV-induced *ddb2* expression results from transcriptional activation accompanied by stabilization of the *ddb2* mRNA, in *P*. *andruzzii* UV induces *ddb2* expression exclusively via an increase in mRNA stability. Thus, we reveal plasticity in the transcriptional and post transcriptional mechanisms regulating the repair of sunlight-induced DNA damage under long-term environmental challenges.

## Author summary

The integrity of genetic information is frequently challenged by environmental factors such as sunlight which induce mutations in DNA. Therefore, DNA damage repair mechanisms are ubiquitous and highly conserved. While significant progress has been made in understanding how these mechanisms recognize and repair DNA damage, how they adapt to long-term environmental challenges remains poorly understood. Cavefish have proven to be fascinating models for exploring the function of DNA repair systems in extreme photic environments. We have previously shown that during evolution for millions of years in complete isolation from sunlight, the Somalian cavefish, *Phreatichthys andruzzii* has lost photoreactivation, a ubiquitous, light-dependent DNA repair system. This results in part from a loss of light, UV and ROS-induced gene transcription. Have other repair systems targeting UV-induced DNA damage been affected in cavefish? Here, we provide evidence that Nucleotide Excision Repair (NER) function is retained in cavefish and is upregulated upon sunlight exposure. Furthermore, we reveal complexity in the transcriptional and posttranscriptional mechanisms regulating the repair of UV-induced DNA damage.

## Introduction

The integrity of DNA is fundamental for the survival of living systems. However, this is frequently challenged by exposure to environmental factors which induce covalent modifications in the structure of DNA and thereby represent a potential source of mutations. For this reason, mechanisms which are able to recognize and repair sites of DNA damage are ubiquitous and appear to have evolved very early during life on the Earth [1–3]. In vertebrates, several DNA repair systems including nucleotide excision repair (NER), base excision repair (BER) and photoreactivation, operate in concert to repair a wide range of DNA lesions [4,5]. Interestingly, eutherians including human and mouse, completely lack photoreactivation repair, an additional, efficient and highly conserved mechanism which is catalysed by photolyases and harnesses visible light to repair UV-induced cyclobutane pyrimidine dimers (CPDs) and 6–4 photoproduct (6-4PPs) lesions [4,6]. Thus, one key unanswered question concerns the evolutionary lability of DNA repair systems and how their function adapts to particular environmental stressors.

Instead of photoreactivation, eutherians rely on the more complex and less efficient NER system to repair UV-induced DNA damage. NER is a major DNA repair system which removes a broad spectrum of DNA helix-distorting lesions, including UV-induced photoproducts as well as bulky base adducts induced by other types of genotoxic stress such as various chemical carcinogens [7–11]. NER is a sequential, multi-step process relying upon tightly coordinated interactions between a set of different proteins [8,12]. One key primary step is how the NER repair machinery identifies the location of DNA lesions within the genome. The heterodimeric DDB1/ DDB2 protein complex (DDB, DNA damage-binding complex) together with the XPC protein (Xeroderma Pigmentosum, Complementation group C) binds to DNA lesion sites with very high affinity, and thereby guides the function of global genomic NER repair [13,14]. Among these factors, DDB2 plays a critical role in the translocation of the whole DDB complex into the nucleus of damaged cells [15–17]. Furthermore, within the general context of the cellular response to DNA damage, DDB2 has also been proposed to function as a transcriptional activator and to interact with cell cycle regulatory proteins [15]. Thus, DDB2 constitutes a key regulatory target in the NER response to environmental stress. Transcriptional regulation of the *ddb2* gene in response to various environmental stressors appears to represent a key determinant of the functionality of this protein [18]. In mammals the expression of *ddb2* is induced by various genotoxic agents, including UV and ionizing radiation [19]. Differently, in fish, *ddb2* as well as *xpc* expression is upregulated upon direct exposure of cells to visible light [20–22]. However, precisely how environmental stressors regulate the expression of these NER factors, as well as how and why this fundamental response has changed during vertebrate evolution remains poorly understood.

The Somalian cavefish, *Phreatichthys andruzzii*, represents an attractive model to study how the function of DNA repair regulatory mechanism is shaped in extreme environments. This species exhibits an extreme, “troglomorphic” phenotype which includes complete loss of the eyes and body pigmentation, an abnormal, blind circadian clock, as well as metabolic adaptations for surviving restricted food availability [23–26]. Geological evidence suggests that this species has evolved completely isolated from sunlit surface water for at least 3 million years when any remaining surface populations were eliminated by desertification. Based on the molecular evolution of cavefish nonvisual photoreceptors, it has been estimated that the functional constraint on cavefish nonvisual photoreceptors was relaxed at ∼5.3 Myr [24]. This implies a long subterranean history, about half of which was in complete isolation from the surface. Interestingly, we have recently revealed that similar to eutherians, *P*. *andruzzii* also exhibits loss of photoreactivation DNA repair [27]. This is the result of a set of point mutations which generate truncated photolyase proteins as well as significant changes in the regulation of *photolyase* gene expression. Namely, light and UV-induced *photolyase* gene transcription is absent due to loss-of-function of a key ROS-responsive promoter element, the D-box enhancer. Loss of photoreactivation in this cavefish is consistent with the absence of sunlight-induced DNA damage, as well as the lack of light to drive photolyase DNA repair function in the constant dark cave environment. However, other factors such as changes in metabolic activity or elevated levels of oxidative stress experienced by cave-dwelling animals [28] may represent an additional source of DNA damage.

Given that NER is able to repair UV damaged DNA and also that expression of the NER recognition factor, *ddb2* is light inducible, we questioned whether NER might also exhibit loss of function in a similar fashion to photoreactivation? To tackle this question, we used a comparative study involving *P*. *andruzzii* and the zebrafish (*Danio rerio*). Our choice of a comparison with zebrafish was based partly on its surface-dwelling habitat. Furthermore, we have extensively studied the transcriptional mechanisms underlying light induced gene expression in this genetic model species [22,27,29–33]. Finally, like *P*. *andruzzii*, the zebrafish is a member of the *Cyprinidae* family and so gene sequences are well conserved between the two species. By comparison of these two species using a comet assay to quantify the presence of DNA strand breaks generated during excision repair, we provide evidence for conservation of NER function in cavefish. Furthermore, the *P*. *andruzzii ddb2* gene coding sequence is highly conserved and in striking contrast to the *photolyase* genes, its expression is robustly induced upon exposure to visible and UV light, as well as reactive oxygen species (ROS) in a similar fashion to the zebrafish. Consistent with the D-box enhancer playing a highly conserved and coordinating role in the DNA repair response in zebrafish, two D-box elements in the *ddb2* promoter are necessary and sufficient to induce *ddb2* expression by visible light and ROS. Surprisingly, also in cavefish the inducibility of the cavefish *ddb2* gene relies upon D-box enhancers, but in cooperation with a proximal E2F binding site. Interestingly, in the case of activation by UV, while zebrafish *ddb2* expression is reliant on transcriptional induction mediated by the D-box enhancers as well as post-transcriptional mRNA stabilization, in the cavefish only *ddb2* mRNA stabilization is observed. *ddb2* gene regulation contrasts with that of the *photolyase* genes, where both UV-induced transcription and transcript stability observed in the zebrafish are completely absent in *P*. *andruzzii* cells. Thus, by comparing the surface-dwelling zebrafish and a cavefish species that has been isolated for millions of years in constant darkness we reveal multiple sunlight-induced transcriptional and post transcriptional mechanisms regulating the repair of UV-induced DNA damage.

## Results

### Conservation of Nucleotide Excision Repair in cavefish *P*. *andruzzii*

**Fig 1:**
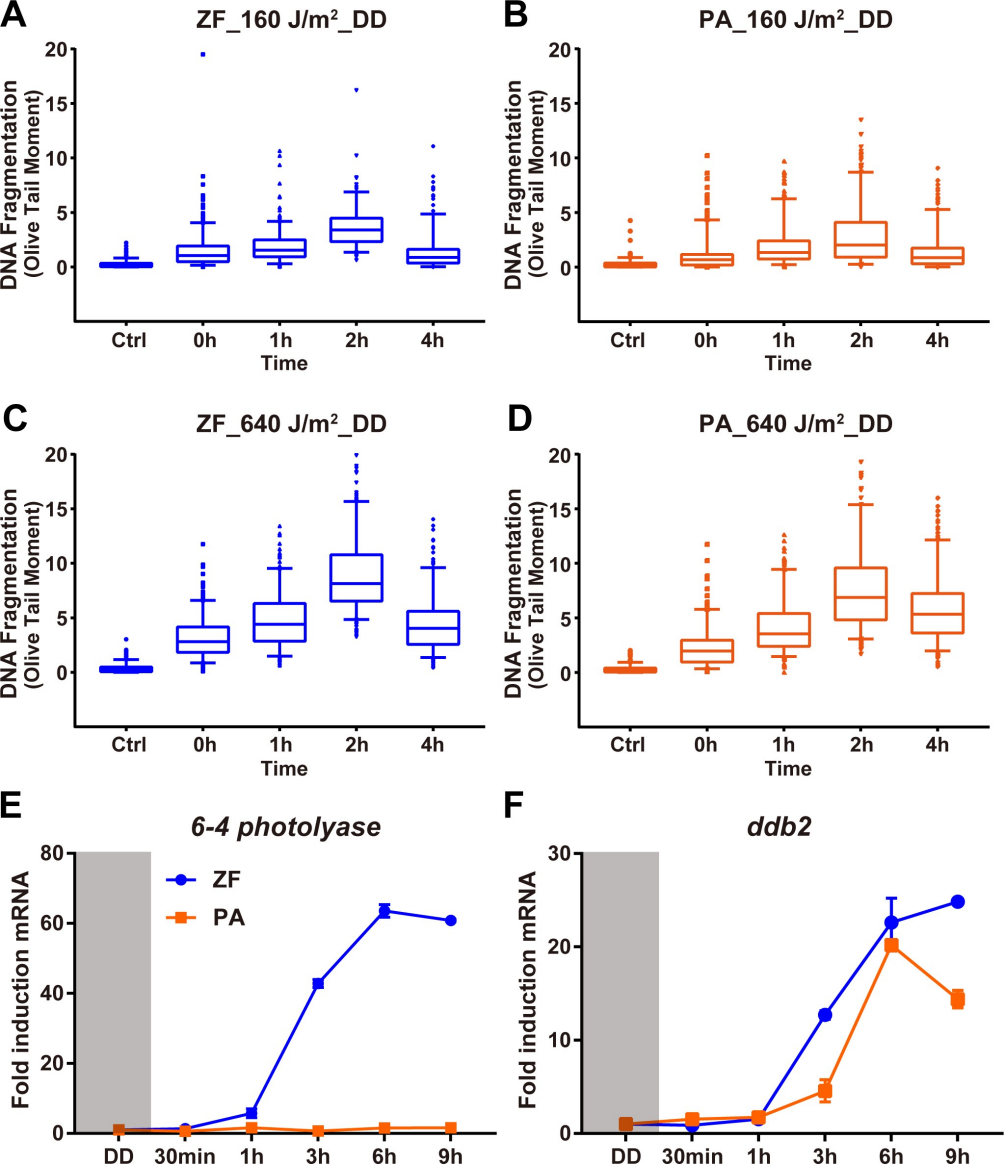
DNA repair of UV induced damage in constant darkness. **(A-D)** Comet assay results from zebrafish AB-9 (A, C) and *P*. *andruzzii* CF-1 (B, D) cells. Data for levels of DNA fragmentation (Olive Tail Moment) is represented as box plots. The median is given as the central line, with 25th and 75th percentiles as frames. Whiskers indicate the 10th and 90th percentiles. Single outliers are illustrated as dots. Times after UV treatment are indicated on the x axes. (n = 3 experimental units, N = 300 observational units per time point). For each panel, Kruskal-Wallis test followed by Dunn’s multiple comparisons test results are reported in S1 Table. **(E,F)** Visible light-induced expression of *ddb2* in zebrafish and *P*. *andruzzii* cells. qRT-PCR analysis of *6–4 photolyase* and *ddb2* mRNA expression in zebrafish PAC-2 (ZF, blue traces) and *P*. *andruzzii* EPA (PA, orange traces) cells during 9 hours of blue light exposure. In each panel, mRNA expression fold induction is plotted on the y-axes as means ± s.d. (n = 3) and times (h and min) are plotted on the x-axes. Each experiment was performed a minimum of three times. Statistical analysis is reported in S1 Table.

We have demonstrated that photoreactivation DNA repair and specifically, *photolyase* function has been lost in cavefish during evolution in constant darkness. The Nuclear Excision repair mechanism (NER) also plays a major role in the repair of UV-induced DNA damage. Therefore, is NER function also affected in this cavefish species? To tackle this question, we assayed DNA repair in cavefish and zebrafish cells following exposure to different doses of UV radiation in complete darkness where photoreactivation repair by *photolyase* should not be active. Specifically, we chose to test the levels of DNA strand break formation, which are generated during nucleotide excision-based DNA repair, by performing a single cell electrophoresis assay, the “comet assay” [34,35]. Contrary to what we previously reported for the photoreactivation DNA repair mechanism, the levels of fragmented DNA following UV exposure (from 40 J/m^2^ to 640 J/m^2^) in both zebrafish [36] and cavefish [25] cells increased, reaching a peak 2 hours after UV exposure and then decreasing at 4 hours in a UV dose dependent manner, with similar induced levels of fragmented DNA in the two cell types (Fig 1A–1D). These results point to comparable levels of NER activity in cavefish and zebrafish cells in response to UV-induced DNA damage.

### Light inducible *ddb2* transcription in cavefish *P*. *andruzzii*

We next wished to explore in more detail the regulation of NER activity in *P*. *andruzzii* and the surface-dwelling zebrafish. We compared the expression of the key NER recognition factor, DDB2, in the two species. We initially cloned and sequenced the *ddb2* transcript from *P*. *andruzzii* and aligned this with the zebrafish ortholog. Consistent with both species being members of the cyprinid family, this revealed a high degree of amino acid sequence conservation (S1 Fig, 79.18%) with no premature truncation or loss of function mutations being evident in cavefish DDB2. However, previous work has shown that *ddb2* basal expression levels are significantly elevated in blind forms of another cavefish species, *Astyanax mexicanus* compared with the surface sighted forms, potentially pointing to increases in *ddb2* expression representing an adaptation for survival in the cave environment [21]. We therefore decided to compare the transcriptional control of this key NER recognition factor in *P*. *andruzzii* and zebrafish. We first observed that *ddb2* mRNA levels were strongly induced by exposure to blue light in both cavefish and zebrafish cell lines. These results are different from our previous study where blue light inducibility was absent in the *photolyase* and other DNA repair genes of *P*. *andruzzii* [27] (Fig 1E and 1F) implying plasticity in how light regulates DNA repair gene expression.

### Differential transcriptional control of the *ddb2* gene in zebrafish and the cavefish *P*. *andruzzii*

**Fig 2:**
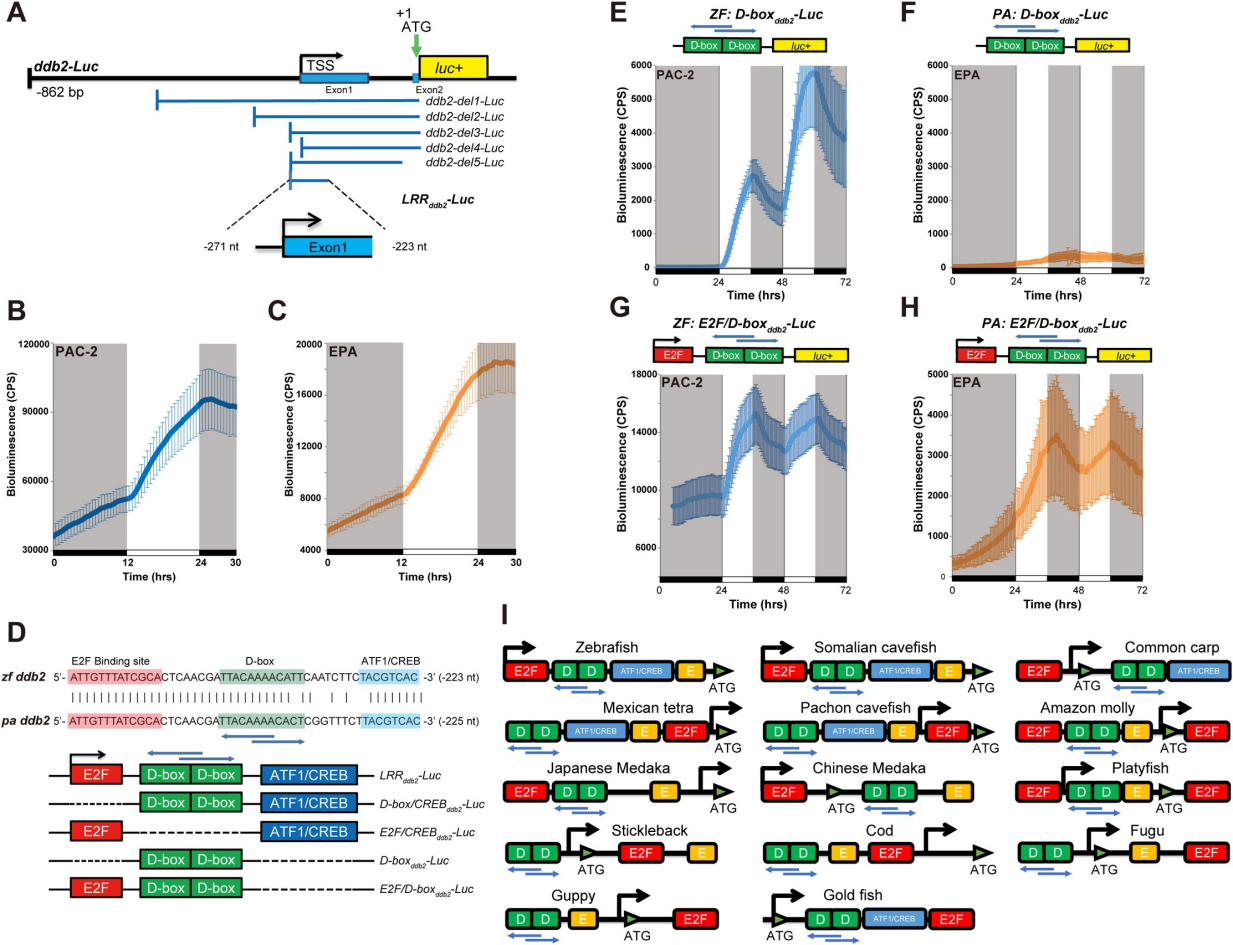
Light-responsive region (LRR) of the *ddb2* promoter. **(A)** Above, schematic representation of the zebrafish *ddb2-Luc* promoter reporter construct. The transcription start site (TSS), ATG translation initiation codon and the luciferase reporter gene (*luc+*) are indicated. Below, schematic representation of the various zebrafish *ddb2* promoter deletion, luciferase reporter constructs analysed. Below is shown the portion of the promoter that constitutes the 49bp LRR_*ddb2*_ region within the *LRR*_*ddb2*_*-Luc* reporter construct. **(B,C)** Representative real-time bioluminescence assays in PAC-2 (B) or EPA (C) cells transfected with the *ddb2-Luc* luciferase reporter. Bioluminescence, counts per second (CPS), is plotted against time (hrs). Black and white bars along the x-axes show dark and light periods, respectively. Each time-point represents the mean of n = 8 ± s.d. **(D)** Sequence alignment of the LRR promoter region in the zebrafish (*zf*) and *P*. *andruzzii* (*pa*) *ddb2* genes. The E2F, D-box and ATF1/CREB enhancer elements are highlighted in red, green and blue respectively. Vertical bars denote identical sequences in the alignment. Blue horizontal arrows indicate the orientation of the two D-box enhancers. The locations of the aligned sequences in terms of nucleotides upstream from the ATG translation start codon (nt) are indicated on the right side of each sequence (in parentheses). Below: schematic representation of the set of LRR_*ddb2*_ sub-deletion constructs where each of the enhancer elements in the *LRR*_*ddb2*_*-Luc* reporter is deleted. The E2F, D-box and ATF1/CREB enhancer elements are represented by red, green and blue boxes respectively. **(E,F)** Above: Schematic representation of the ZF and PA sequence *D-box*_*ddb2*_*-Luc* reporters. Below: Representative real-time bioluminescence assays from zebrafish PAC-2 (blue trace, left panel) and cavefish EPA cells (orange trace, right panel) transfected with ZF: and PA: *D-box*_*ddb2*_*-Luc* respectively and exposed to light and dark periods. Bioluminescence (CPS) is plotted on the y-axes and time (hrs) on the x-axes. Each time-point represents the mean of n = 8 ± s.d. White and black bars below each panel represent the light and dark periods, respectively. **(G,H)** Above: Schematic representation of the ZF and PA *E2F/D-box*_*ddb2*_*-Luc* reporter constructs. Below: Representative real-time bioluminescence assays from PAC-2 (left panel) and EPA cells (right panel) transfected with ZF: and PA: *E2F/D-box*_*ddb2*_*-Luc* respectively and exposed to light and dark periods (as described for panel E-F). **(I)** Schematic representation of the E2F site, D-box and E-box elements as well as ATF1/CREB site present in the promoters of *ddb2* genes in various fish species. These elements are conserved in a broad range of fish species (see S2 Table for the Ensembl accession numbers).

Which transcription control mechanisms underlie the differences observed between the *photolyase* (Photoreactivation*)* and *ddb2* (NER) DNA repair genes? We previously showed that in the case of the zebrafish *photolyase* genes, the functionality of two D-box enhancer elements is necessary and sufficient to activate transcription [27]. Here, we initially cloned an 862 bp fragment of the 5´ region of the zebrafish *ddb2* gene into a luciferase reporter vector (*ddb2-Luc*) (Fig 2A). Transient transfection of the *ddb2-Luc* reporter construct into both zebrafish and *P*. *andruzzii* cells exposed for 12 hours to blue light after an extended period in darkness, revealed a light-driven induction of bioluminescence in both cell types (Fig 2B and 2C). This result is consistent with the endogenous *ddb2* gene expression (Fig 1F) and indicates that this construct contains all the regulatory sequence elements required to direct light-induced gene expression.

In order to identify the minimal region of the zebrafish *ddb2* promoter able to direct light-inducible gene expression, we performed an unbiased promoter analysis using a series of overlapping promoter deletion constructs transfected in both zebrafish PAC-2 and cavefish EPA cells which were then exposed to light (Figs 2A and S2A–S2H). Our results defined a minimal 49 bp fragment (termed Light Responsive Region, LRR_*ddb2*_) that was sufficient to drive light-induced transcription in both cell types (Figs 2A–2D and S2H). The LRR_*ddb2*_ contains D-box, E2F and ATF1/CREB consensus enhancer sequences that are highly conserved between the zebrafish and cavefish *ddb2* genes (Fig 2D). Consistent with our previous description of the *photolyase* genes [27], the analysis of constructs carrying specific deletions of each enhancer sequence within the zebrafish LRR_*ddb2*_ region (Fig 2D), revealed that the two D-box enhancer elements lying immediately downstream of the predicted transcription start site (TSS) were essential and sufficient for directing light-induced expression in zebrafish cells (Figs 2E and 2G and S2H–S2L). However, in cavefish cells light-induced *ddb2* expression was only conferred by these two D-box enhancers when in combination with the proximal E2F binding site within the cavefish LRR_*ddb2*_ region (Figs 2F–2H and S2H–S2L). Thus, the D-box enhancer plays a key role in mediating light-driven transcription of a DNA repair gene in both zebrafish and *P*. *andruzzii* cells. Furthermore, in the case of *ddb2* in *P*. *andruzzii* cells, D-box function relies upon cooperation with an adjacent E2F enhancer element.

How conserved is the role of the E2F&D-box enhancers in regulating expression of the *ddb2* gene in other fish species? To address this question we next scrutinized the *ddb2* promoter sequences in a broad range of fish genome sequences (see S2 Table). Our results reveal that the D-box enhancer element as well as the E2F site are present in all the *ddb2* promoter regions analysed, in close proximity to their transcription start sites (Fig 2I and S2 Table). These findings are consistent with the central role for the D-box in fish species as a target for light-dependent signalling. Furthermore, E2F function has also been implicated in the regulation of *ddb2* expression in mammals [37,38]. Specifically, the E2F binding site has been proposed to serve as a *ddb2* basal expression activator in mammalian cells. Consistent with the notion that E2F may serve a similar function in fish cells, *E2F/D-box*_*ddb2*_*-Luc* reporter (including the E2F site) expression levels were significantly higher than those of the *D-box*_*ddb2*_*-Luc* reporter (lacking the E2F site) during exposure to light-dark cycles (S3 Fig, panel A). Thus, our data point to an evolutionary highly conserved function for E2F and the D-box enhancer in regulating *ddb2* expression.

### Regulation of light-driven *ddb2* expression via ROS signalling

**Fig 3:**
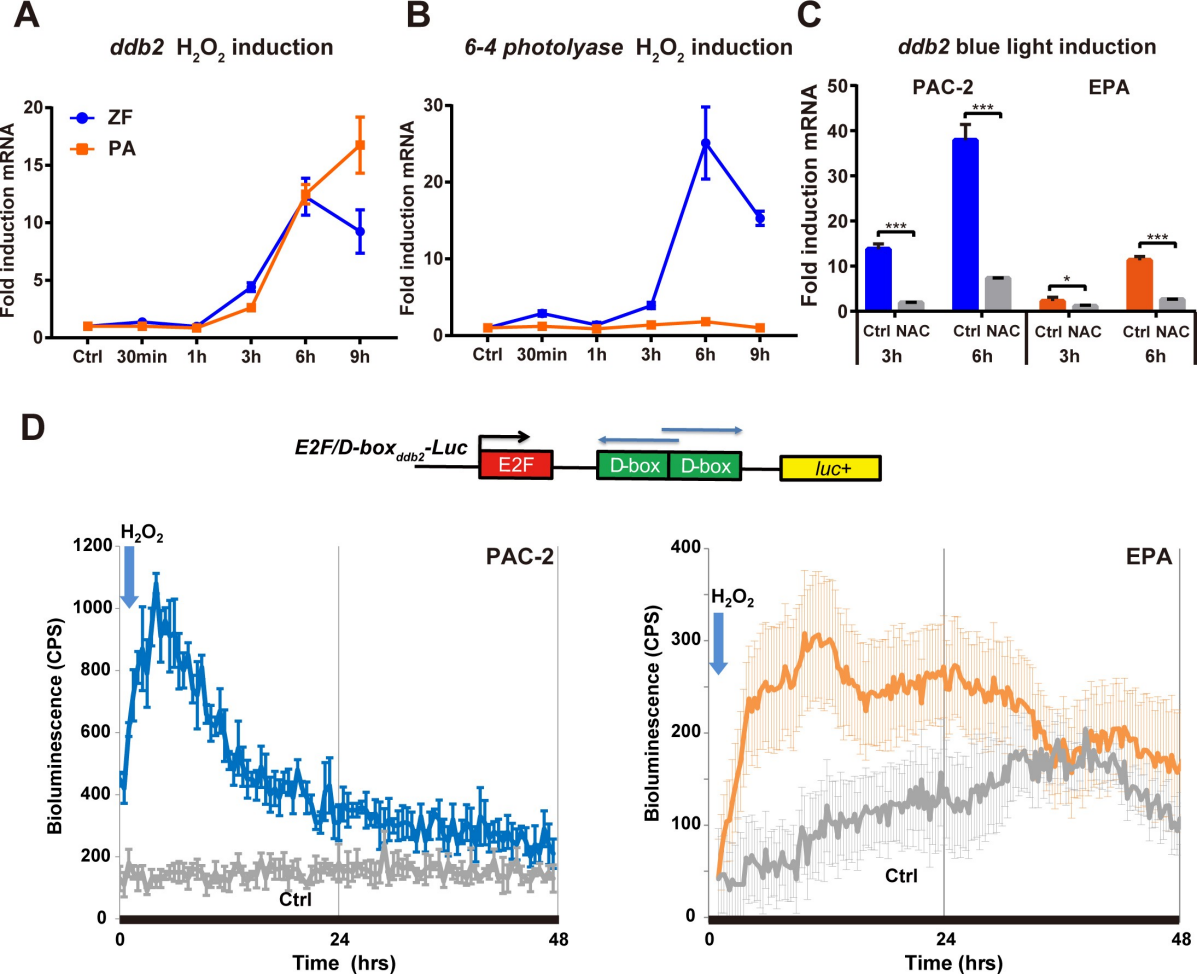
Light regulation of *ddb2* expression via ROS signalling. **(A,B)** qRT-PCR analysis in zebrafish PAC-2 (blue traces) and cavefish EPA (orange traces) cells after 300 μM H_2_O_2_ treatment. Relative mRNA expression for *ddb2* (panel A) and *6–4 photolyase* (panel B) are plotted on the y-axes as mean (n = 3) ± s.d.. Times (h and min) are plotted on the x-axes. Two-way ANOVA followed by Sidak’s multiple comparisons test results are reported in S1 Table. **(C)** qRT-PCR analysis in zebrafish and cavefish cells treated with 6 mM N-acetylcysteine (NAC) or vehicle (Ctrl), following 3 or 6 hours exposure to blue light. On the y-axes are plotted the fold induction (± s.d.) of expression with respect to samples subjected to identical treatment but maintained under DD. Times(hrs) are indicated on the x axes. Statistical analysis results (Student’s t-test (unpaired, two tailed)) are represented by asterisks (***p<0.001, **p<0.01, *p<0.05) and reported in S1 Table. **(D)** Representative real-time bioluminescence assays from zebrafish PAC-2 (blue trace, left side) and cavefish EPA (orange trace, right side) cells transfected with the *E2F/D-box*_*ddb2*_*-Luc* reporter (above) and treated in DD with 300 μM H_2_O_2_ at the time points indicated by the blue arrows. Grey traces represent control transfected PAC-2 and EPA cells that were not treated with H_2_O_2_. Bioluminescence, counts per second (CPS), is plotted against time (hrs). Each time-point represents the mean of n = 8 ± s.d. Each experiment was performed a minimum of three times.

Which signalling pathways link transcription of the *ddb2* gene with light exposure in zebrafish and cavefish cells? We have recently implicated an increase of reactive oxygen species (ROS) levels as a prerequisite for light-induced gene expression via the D-box enhancer in zebrafish [27,29]. Furthermore, ROS-activated gene expression via the D-box appears to coordinate the gene expression response to oxidative stress [29]. Consistent with ROS serving as a key regulatory signal for *ddb2* expression in both zebrafish and cavefish cells, endogenous *ddb2* gene expression was robustly induced in both zebrafish PAC-2 (blue traces) and cavefish EPA (orange traces) cells upon treatment with H_2_O_2_ (Fig 3A). This again contrasts with the expression of the *6–4 photolyase* gene, which is induced in zebrafish but not cavefish treated cells (Fig 3B and [27]). Furthermore, pre-treatment with N-acetylcysteine (NAC), a general ROS scavenger, efficiently attenuated blue light-induced *ddb2* gene expression in both zebrafish and cavefish cells (Fig 3C) pointing to a ROS-dependent, light-responsive mechanism also acting in cavefish cells. Similar to the pattern observed for endogenous *ddb2* gene expression, H_2_O_2_ treatment successfully activated *E2F/D-box*_*ddb2*_*-Luc* reporter expression in transfected zebrafish PAC-2 and cavefish EPA cells (Fig 3D). In addition, consistent with the notion that the E2F site serves as a *ddb2* basal expression activator, expression levels of the *E2F/D-box*_*ddb2*_*-Luc* reporter (including the E2F site) were significantly higher than those of the *D-box*_*ddb2*_*-Luc* reporter (lacking the E2F site) upon treatment with H_2_O_2_ (S3 Fig, panel B). Thus, our results point to the E2F site and D-boxes serving as the convergence point for the mechanisms directing ROS-induced *ddb2* gene expression in cavefish *P*. *andruzzii* cells.

### UV-regulated, post-transcriptional control of *ddb2* expression

**Fig 4:**
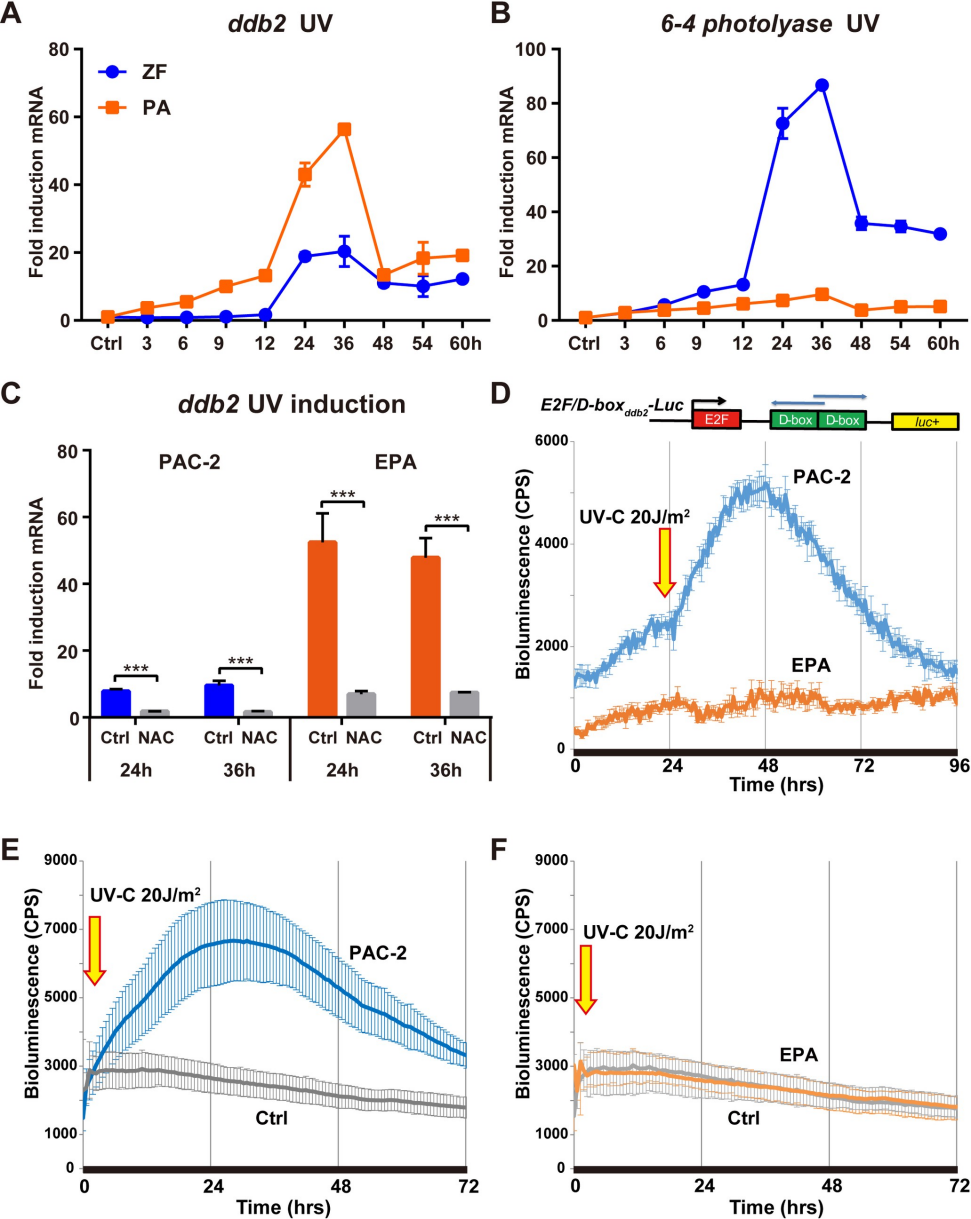
UV responsive *ddb2* gene expression. **(A,B)** qRT-PCR analysis of *ddb2* (A) and *6–4 photolyase* (B) mRNA expression in PAC-2 (ZF) and EPA (PA) cells during a 60-hour period in DD following exposure to a short UV-C pulse (20 J/m^2^). **(C)** qRT-PCR analysis in zebrafish and cavefish cells treated with 6 mM N-acetylcysteine (NAC) or vehicle (Ctrl), at 24 hours and 36 hours following a short UV-C pulse (20 J/m^2^). On the y-axes are plotted the fold induction (± s.d.) of expression with respect to samples subjected to identical treatment but maintained under DD. Times are indicated on the x-axes. Statistical analysis results (Student’s t-test (unpaired, two tailed)) are represented by asterisks (***p<0.001, **p<0.01, *p<0.05) and reported in S1 Table. **(D)** Bioluminescence analysis of PAC-2 (blue trace) and EPA (orange trace) cells transfected with the *E2F/D-box*_*ddb2*_*-Luc* reporter, maintained in constant darkness following exposure to a short UV-C light pulse (20 J/m^2^). **(E,F)** Comparable bioluminescence analysis of PAC-2 (blue trace) and EPA (orange trace) cells transfected with the *ddb2-Luc* reporter, maintained in constant darkness following exposure to a short UV-C pulse (20 J/m^2^). Grey traces in panels E and F represent control (Ctrl) transfected PAC-2 and EPA cells, respectively, that were not treated with UV-C. Each experiment was performed a minimum of three times.

We have recently reported that exposure to UV radiation, as well as generating DNA damage also induces the transcription of the *photolyase*, *neil1* and *xpc* DNA repair genes in zebrafish. This property is absent in cavefish cells, possibly the consequence of evolution in the complete absence of UV radiation in the cave environment [27]. We therefore questioned whether, as for the response to visible light and ROS, *ddb2* might retain UV inducibility in cavefish cells. Therefore, we performed real-time qPCR analysis of *ddb2* expression in zebrafish and *P*. *andruzzii* cells over a 60-hour period in constant darkness following acute exposure (13 seconds) to a UV-C pulse of 20 J/m^2^, assaying *6–4 photolyase* mRNA expression as a control. In contrast to the situation observed for the cavefish *6–4 photolyase* gene, both cavefish and zebrafish *ddb2* genes showed robust induction following acute UV exposure (Fig 4A and 4B). Pre-treatment with N-acetylcysteine (NAC) efficiently blocks UV-induced *ddb2* gene expression in both zebrafish and cavefish cells (Fig 4C), pointing to the UV-induced elevation of cellular ROS levels being a prerequisite for *ddb2* transactivation in both fish species. Thus, in contrast to other DNA repair genes, the *ddb2* gene retains inducibility by visible light, ROS as well as UV exposure in cavefish cells.

To address whether the E2F/D-box_*ddb2*_ elements identified in the light and ROS responsive region in cavefish cells might also be involved in the UV-inducibility of *ddb2* gene expression, we measured bioluminescence of the *E2F/D-box*_*ddb2*_*-Luc* reporter construct in transfected zebrafish and cavefish cells exposed to UV light (Fig 4D). While in zebrafish cells this reporter was robustly induced with kinetics resembling those of the endogenous *ddb2* gene (Fig 4D, blue trace), surprisingly, the expression of *E2F/D-box*_*ddb2*_*-Luc* was not induced in UV-treated cavefish cells (Fig 4D, orange trace). This result in cavefish cells contrasts with the strong induction of endogenous *ddb2* gene expression previously observed (Fig 4A).

To test the possibility that elements other than the E2F/D-boxes in the *ddb2* promoter region might confer UV inducibility in cavefish, we assayed expression of the full 862bp promoter construct (*ddb2-Luc*) in response to a UV pulse (20 J/m^2^) in both types of cells. Consistent with our previous result, the bioluminescence from this reporter was strongly induced following an UV-C pulse in zebrafish cells (Fig 4E) but not in transfected cavefish EPA cells (Fig 4F), pointing to the absence of UV-inducible enhancer elements in proximity of the start site of transcription for the cavefish gene.

### UV increases DNA repair gene mRNA stability

**Fig 5:**
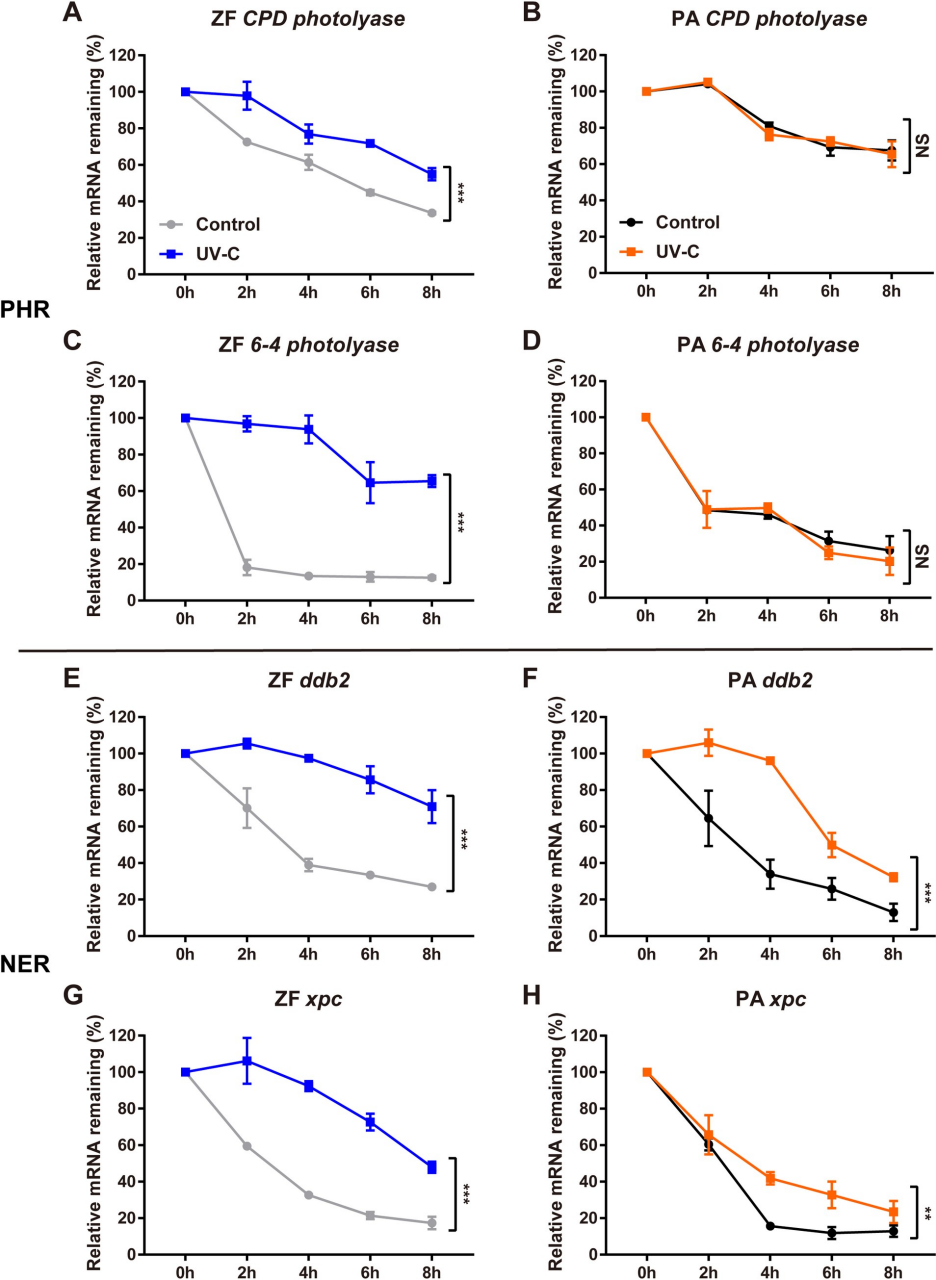
mRNA stability of DNA repair genes following UV radiation. qRT-PCR analysis of *CPD photolyase* (A,B), *6–4 photolyase* (C,D), *ddb2* (E,F) and *xpc* (G,H) mRNA expression levels in PAC-2 (left panels, ZF) and EPA (right panels, PA) cells exposed to a UV-C pulse (20 J/m^2^), then maintained under constant darkness for 24 hours followed by treatment with actinomycin D (5 μg/ml). On the y axes, relative remaining levels of mRNA (% ± s.d. compared with levels at the onset of the actinomycin D treatment) are plotted (blue or orange traces, UV-C). The results from control samples subjected to actinomycin D treatment and held for the same period in constant darkness but without UV radiation are plotted on the same axes (grey or black traces), in order to compare the mRNA decay for these genes in the presence and absence of on-going transcription. The time points at which samples were taken following the onset of actinomycin D treatment (h) are indicated on the x axes. Statistical analysis of the results is represented by asterisks (***p<0.001, **p<0.01, *p<0.05) and reported in S1 Table.

Which mechanisms may account for the differences between endogenous and exogenous *ddb2* expression following the exposure of cavefish cells to UV? While promoter reporter assays document levels of transcription, endogenous gene expression levels reflect both transcriptional and post-transcriptional control, including changes in mRNA stability. It has been reported that environmental stressors such as UV exposure activate gene expression at multiple levels, involving transcriptional as well as post-transcriptional regulatory mechanisms [39]. Specifically, in mammals recent studies have demonstrated that the direct or indirect effects of UV exposure serve to stabilize the *ddb2* transcript and thereby contribute to its accumulation following UV treatment [40,41]. Thus, our results may be consistent with the presence of a non-transcriptional mechanism mediating the UV-induced expression of *ddb2* in the cavefish *P*. *andruzzii*. We therefore decided to investigate the influence of UV exposure on stability of the *ddb2* as well as the *photolyase* transcripts in zebrafish and *P*. *andruzzii* cells (Fig 5). Cells were exposed to a pulse of UV-C (20 J/m^2^) and then during the subsequent period in complete darkness, they were treated with the transcription inhibitor, actinomycin D. We then used qRT-PCR to assay the declining levels of specific mRNAs and thereby to visualize their decay rate. Interestingly, in zebrafish cells we observed an UV-induced increase in mRNA stability in the case of all DNA repair genes (Fig 5, left panels) indicating that the rate of mRNA turnover for these zebrafish transcripts was significantly reduced following UV-C exposure. In contrast, no significant difference in the rate of mRNA decline between the UV-treated and control groups was observed in cavefish cells for the *photolyase* genes (Fig 5B–5D), while the *ddb2* gene transcript was significantly stabilized following UV exposure as in zebrafish cells (Fig 5F). This is consistent with the increase of endogenous *ddb2* transcript levels observed following UV treatment (see Fig 4A). Interestingly, we observed a similar increase in transcript stability of the other key NER regulatory factor XPC in both zebrafish and cavefish cells (Fig 5G and 5H), pointing to a general role for increased transcript stability in the upregulation of NER function following UV exposure. Thus, our results point to the conservation of normal NER function in cavefish as the result of gene-specific differences in both transcriptional and post-transcriptional regulation of gene expression.

## Discussion

In this study, we provide insight into how a perpetually dark environment, completely isolated from the damaging effects of UV radiation, differentially shapes the DNA repair response in cave organisms. In our previous studies we have shown that the Somalian cavefish *Phreatichthys andruzzii* lacks photoreactivation repair in part due to premature truncating, loss of function mutations affecting a subset of photolyases. Furthermore, in this species, *photolyase* genes completely lack light, UV and ROS-inducible transcription [27]. Here in contrast, we demonstrate that the NER system which also repairs UV damaged DNA, is conserved in this cavefish. The expression of *P*. *andruzzii ddb2*, a key recognition factor in NER DNA repair, displays the normal visible light, UV and ROS-inducible expression pattern observed in zebrafish. However, we reveal that the precise nature and combination of transcriptional and post-transcriptional mechanisms inducing *ddb2* expression in the cavefish differ from those in the zebrafish. Furthermore, these mechanisms differ from those regulating the *photolyase* genes. Our findings provide valuable additional insight into the general mechanisms whereby light, UV and ROS exposure trigger changes in gene expression. Furthermore, we reveal how multiple transcriptional and posttranscriptional regulatory mechanisms differentially shape DNA repair system function under extreme environmental conditions.

### Species-specific regulation of the *ddb2* gene

Our results point to a surprising degree of flexibility in the transcription control mechanisms for *ddb2*, within different fish species. The integrity of two D-box enhancers is essential for visible light, UV and ROS regulated *ddb2* gene expression in zebrafish. However, in *P*. *andruzzii* the conserved D-box sequences cooperate with an adjacent E2F site to confer visible light and ROS inducibility. Interestingly, an E2F binding site has been proposed to serve as a *ddb2* basal expression activator in mammalian systems [37,38]. In contrast, neither the D-boxes nor the E2F site are responsible for the induction of *ddb2* expression in the cavefish following UV light exposure. Consistent with an inherent flexibility in the regulation of this key NER recognition factor during vertebrate evolution, in humans, p53 plays a key role in directing UV inducible expression of *ddb2*, with a functional p53 response element being identified in its promoter region [42–44]. However in mouse, p53 fails to transactivate *ddb2* expression following UV irradiation [45]. UV radiation can induce *ddb2* transcription via D-box enhancer elements in zebrafish and also we were unable to identify consensus p53 binding sites in the zebrafish or cavefish *ddb2* promoter regions. Therefore, the regulatory role of p53 in controlling *ddb2* expression may have been replaced by D-box binding transcription factors in this species, while in the cavefish, *ddb2* seems to respond to UV and DNA damage via a p53- and D-box enhancer-independent pathway. Given the central role played by DDB2 in sensing DNA damage and coordinating the NER response, it is tempting to speculate that the flexibility of its gene expression control mechanisms may serve to tailor DDB2 function to optimally counter the unique set of environmental challenges encountered by each species.

Our characterization of *ddb2* regulation in zebrafish and blind cavefish provides new insight into the mechanisms whereby light exposure regulates gene expression in fish. In our previous studies we have pinpointed the D-box enhancer as mediating light-induced gene expression of clock genes (*per2* and *cry1a*) [30,31] and as well as the *photolyase* genes [27]. Furthermore, in the cavefish *P*. *andruzzii*, we have revealed that loss of D-box function underlies the absence of light-induced expression of many genes in this species. However, the prediction that a single regulatory mechanism based on the D-box may direct light responsive gene expression is clearly challenged by our observation that *P*. *andruzzii ddb2* expression is still strongly light inducible. Clearly, not all genes that are light inducible have lost this functionality in *P*. *andruzzii*, an observation hinting at complexity at the level of D-box regulation. Interestingly this enhancer element represents the binding site for a complex repertoire of homo and heterodimers of PAR bZip factors and E4BP4 / nfil3 bZip factors [20,46–48]. It is possible that interaction of the D-box enhancer with other enhancers such as the E2F site may influence the types of factors binding to the D-box. In this scenario, certain D-box regulatory factors which do retain light responsive regulatory function in *P*. *andruzzii*, are able to bind and regulate transcription. Instead, in other genes such as the *photolyase* genes, distinct combinations of factors regulate the D-box which are no longer light responsive.

### Evolution of post transcriptional response to UV exposure

Our study has implicated post-transcriptional regulation playing a role in upregulating DNA repair gene expression upon UV radiation exposure. Furthermore, this mechanism appears to demonstrate species and gene-specific flexibility, with loss of UV induced mRNA stability in the case of the *photolyase* genes but not the genes encoding the NER factors *ddb2* and *xpc* in the blind cavefish *P*. *andruzzii*. Although transcription has been the focus of efforts to understand the regulation of gene expression, mRNA stability has also emerged as an important regulatory target for environmental stress and endogenous mediators [49–51]. For example, previous studies have analysed changes in mRNA stability in human cell lines which had been subjected to stress-inducing agents. Strikingly, ~50% of the affected transcripts showed altered abundance due to changes in mRNA stability rather than changes in transcription [52]. Central to the regulation of mRNA stability are cis-regulatory sequences in the non-coding regions of mRNAs as well as key RNA binding factors. An important group of regulatory mRNA elements are AU-rich elements (AREs), which are found in the 3’-UTRs of many rapidly inducible genes with high mRNA decay rates [50,53].

While there have been no previous reports of how mRNA stability contributes to the regulation of *photolyase* gene expression, the *ddb2* transcript has been documented to exhibit rapid turnover [54], with a short region within its 3’ UTR affecting its transcription and decay [41]. This 3’ UTR sequence which does not include ARE sequences, is recognized as co-transcriptionally linking transcriptional and post-transcriptional regulatory pathways. Furthermore, similar sequences were identified in the 3’ UTRs of unrelated transcripts (ZNF493, SS18, MAK and XPNPEP3), suggesting that the post-transcriptional control mechanism targeting this element may be involved in the coordinated regulation of multiple genes [41]. Indeed, previous data has indicated that UV light exerts a strong stabilizing effect on a range of different mRNAs that are associated with 3’-UTR sequences that do not contain AREs [39].

The mechanism of UV-induced stabilization remains unidentified. One of the direct effects of UV light is the site-specific damage of 28S rRNA [55]. This has been suggested to trigger a ribotoxic stress response which includes translation inhibition as well as activation of stress kinase pathways and gene expression. It is possible that ribosomal damage or its consequences leads to inhibition of mRNA decay mechanisms. We have previously reported that activation of MAPK stress signalling pathway upon exposure of zebrafish cells to UV radiation represents an essential trigger for inducing the transcription of both DNA repair and clock genes [27]. Furthermore, activation of stress MAP kinase pathways has also been linked with changes in mRNA stability. While the p38/MK2 pathway, activated by inflammatory stimuli such as IL-1 and LPS, induces stabilization of various ARE- containing mRNAs, UV light appears to activate a more general mechanism of mRNA stabilization, one that is independent of AREs and the p38/MAP kinase pathway [39]. Therefore, the precise links between the effects of UV exposure, induced stress MAP kinase signalling and the enhancement of mRNA stability remain unclear. Clearly, a future challenge will be to identify the regulatory elements which are responsible for the fundamental gene-specific changes in mRNA stability that have occurred during evolution.

### Evolution of DNA repair pathways

The observed differences in *ddb2* regulation between *P*. *andruzzii* and zebrafish are consistent with other documented species-specific differences in the regulation of this gene. However, additional key factors that must be considered are the strong evolutionary pressures that operate in the cave environment and which result in the profound troglomorphisms observed in cavefish species. The significance of retaining *ddb2* inducibility by visible light and UV radiation exposure in an environment completely isolated from exposure to sunlight is at first sight paradoxical. However, we have established that regulation of gene expression by both visible and UV light in fish cells relies upon light-induced increases in levels of ROS [27,29,56]. Therefore, the continued regulation of *ddb2* by sunlight under artificial experimental conditions may reflect conserved regulation of NER DNA repair by ROS in the actual cave environment. Interestingly, a previous report in the cavefish *Astyanax mexicanus* has shown that the levels of *ddb2* expression are significantly elevated in blind cave forms compared with their surface dwelling, sighted relatives [21]. Furthermore, high basal levels of expression of *CPD photolyase* have also been reported in the blind cave forms of this species [21]. The upregulated *ddb2* and *CPD phr* expression appears to contribute to an enhanced DNA repair capacity upon UV damage [21]. Furthermore, it has been speculated that this might reflect different types of DNA damage experienced in the cave environment and that elevated *ddb2* and *CPD phr* expression may provide a selective advantage in repairing these types of DNA damage. For example, life in the hypoxic and slightly acidic water locked inside these cave systems may lead to an increase of intracellular oxidative stress and thereby elevate DNA damage [57]. Interestingly, an enhancement of DNA repair capacity has also been identified in the blind mole rat, *Spalax*, linked with its hypoxia tolerance [58]. However, countering this argument, in a comparative study using cave and surface populations of the amphipod crustacean *Gammarus minus*, reduced expression of both *photolyase* and the DNA repair endonuclease XPG (ERCC5, a component of the NER pathway) was observed in the cave population, suggesting that all these genes are under relaxed selection in the cave populations [59]. However, a careful analysis of the environmental conditions experienced by these particular cave organisms will be essential before reaching general conclusions. Nevertheless, considering our findings and those from other cavefish species, it is tempting to speculate that alterations to DNA repair systems may represent a common solution to the challenges of life in extreme subterranean and cave environments.

## Materials and methods

### Cell culture

All the fish cell lines utilized were maintained as previously described for the PAC-2 cells [25,60]. The zebrafish embryonic cell line PAC-2 [61], cavefish embryonic cell line EPA [29], zebrafish adult fin cell line AB9 [36] and cavefish adult fin cell line CF1 [25] were cultured in L-15 (Leibovitz) medium (Gibco BRL) supplemented with 15% fetal Calf Serum (Sigma Aldrich F7524), 100 units/ml penicillin, 100 μg/ml streptomycin and 50 μg/ml gentamicin (Gibco BRL). Cells were maintained in an atmospheric CO_2_, non-humidified cell culture incubator at 26°C.

### “Comet assay”: the single cell gel electrophoresis assay

The “Comet Assay” was performed as previously described [27]. Specifically, zebrafish and cavefish cells were cultured according to former reports. For the irradiation experiments, regularly passaged cells were transferred to 6-well plates at a concentration of 5×10^5^ cells/mL, in a total volume of 2 mL per well. Cells were incubated overnight in the dark according to specific culturing conditions. After 24h, cells were exposed to specific doses of UV radiation (160 and 640 J/m^2^), using a UV-strata linker (Stratagene). A non-exposed plate served as negative control. Following irradiation, cells were returned to the incubator and sampled at specific time points (0, 1, 2, and 4h) in quadruplicates. Sampled cells were detached by trypsinization and embedded in 0.7% low-melting agarose on microscope slides and lysed in a slide chamber containing alkaline lysis buffer at 4°C overnight. Subsequently, slides were transferred to the electrophoresis chamber (Bio-Rad), filled with ice-cold buffer. Electrophoresis was conducted at 25 V and 0.3 A for 20 min. Finally, slides were neutralized, stained with ethidium bromide (EtBr) and then imaged using an epifluorescence microscope (Zeiss Axiostar) equipped with a green light excitation filter of 518 nm. Images were analyzed according to the Olive Tail Moment system [62].

### Cloning and gene expression analysis

Total RNA from zebrafish and *P*. *andruzzii* cells was extracted with Trizol Reagent (Gibco, BRL) according to the manufacturer’s instructions. Reverse transcription was performed using M-MLV reverse transcriptase (Thermo Scientific) and then the cDNAs were cloned into the pGEM-T Easy Vector (Promega). Sequencing was performed by Microsynth Seqlab (Göttingen, Germany). For promoter analysis, fragments from the zebrafish *ddb2* gene promoters were amplified by genomic PCR based on database information (ENSDARG00000041140) and subcloned into the luciferase reporter pGL3-basic vector (Promega). Cavefish *ddb2* gene promoter sequences were amplified by genomic PCR using primers based on a partial *P*. *andruzzii* genome sequence. To generate overlapping deletion/mutation constructs, unique enzyme digestion or site-directed mutagenesis by using Q5 Site-Directed Mutagenesis Kit (New England Biolabs) were performed according to the manufacturer’s recommendations. Detailed information on each construct is described in Fig 2 and S3 Table.

For gene expression analysis, cDNA was synthesized from total RNA extracts using M-MLV reverse transcriptase (Thermo Scientific). Quantitative PCR was performed using the Step One Plus Real-Time PCR System (Applied Biosystems) and SYBRGreen (Promega) master mix according to the manufacturers’ recommendations. Primers are shown in S4 Table. The relative expression levels for each gene were calculated by the 2^-ΔΔCT^ method and normalized using the relative expression of *β-actin*.

### mRNA stability analysis

One way of analysing mRNA kinetics involves blocking cellular transcription with inhibitors like actinomycin D (which interferes with transcription by intercalating into DNA and inhibition of DNA-dependent RNA synthesis). Zebrafish PAC-2 and cavefish EPA cells were treated with a 20 J/m^2^ UV-C pulse and incubated under constant darkness for 24 hours (peaking time for UV-induced mRNA expression, [27]). Cells were then treated with 5 μg/ml of Actinomycin D (A1410, Sigma-Aldrich) to block transcription and aliquots were removed periodically. cDNA was synthesized from total RNA extracts using M-MLV reverse transcriptase (Thermo Scientific). qRT-PCR was performed and the relative amount of a particular mRNA remaining at various times of treatment is used to calculate the mRNA decay rate. Primers used for qRT-PCR are reported in S4 Table.

### Real-time bioluminescence assay

Real-time bioluminescence assays were performed as previously described [30,31]. Cells were transfected using Fugene HD reagent (Promega) according to the manufacturer’s instructions. After 24hrs, 0.5 mM beetle luciferin, potassium salt (Promega) was added to the culture medium. Bioluminescence was measured using a Topcount NXT or Envision counter (Perkin Elmer). The data were analysed using the Microsoft Excel “Import and Analysis” macro (S. Kay, Scripps Research Institute).

### Statistical analysis

Data were analyzed using GraphPad Prism 7 (GraphPad Software Inc.). All the results were expressed as means ± standard deviation. Student’s t-test or analysis of variance (ANOVA) were used to determine significant differences followed by multiple comparison post-test. P values< 0.05 were considered statistically significant. Statistical differences of p< 0.05, p< 0.01, p< 0.001 are represented by *, ** or ***, respectively. All detailed statistical information is shown in S1 Table. The original data used to make all the figures and statistical analyses in this study can be found in the S1 Dataset.

## Supporting information

S1 Fig Amino acid sequence alignment of zebrafish and cavefish DDB2. The black fonts denote identical sequences while the red text represent the mismatched amino acids in the sequence alignment. The locations of the aligned sequences are indicated on the right side of each sequence. (The cavefish *ddb2* coding sequence reported in this article has been deposited in GenBank under accession number: Genbank MN907102).
(TIF)

S2 Fig Detailed promoter analysis of the *ddb2* gene. **(A)** Schematic representation of the various zebrafish *ddb2* promoter luciferase reporter constructs analyzed. The position of exon sequences, the transcription start site, ATG translation start codon and the luciferase reporter gene (luc+) are indicated. Below, within the context of the minimal, light-responsive promoter construct *LRR*_*ddb2*_*-Luc* are indicated the E2F site, D-box and ATF1/CREB enhancer elements by red, green and blue rectangles, respectively. Blue arrows above the D-boxes indicate their orientation. **(B-G)** Real-time bioluminescence assays from zebrafish PAC-2 (blue traces) and cavefish EPA cells (orange traces) transfected with the various zebrafish *ddb2* promoter luciferase reporter constructs. Bioluminescence (CPS) is plotted on the y-axes and time (hrs) on the x-axes. Each time-point represents the mean of n = 8 ± s.d.. Black and white bars along the x-axes show dark and light periods. **(H-L)** Real-time bioluminescence assays from PAC-2 (blue traces) and EPA cells (orange traces) transfected with the sub-deletion constructs derived from *LRR*_*ddb2*_*-Luc* where individual enhancers are deleted. Above each panel is a schematic representation of each construct. Bioluminescence data is presented as described for panels B-G.
(TIF)

S3 Fig E2F site plays a role in *ddb2* basal expression. **(A,B)** Above: Schematic representation of the zebrafish *E2F/D-box*_*ddb2*_*-Luc* (left panel) and *D-box*_*ddb2*_*-Luc* (right panel) reporter. **(A)** Below: representative real-time bioluminescence assays from zebrafish PAC-2 cells transfected with corresponding luciferase reporter vector and exposed to 12 hours of light within a period of constant darkness. **(B)** Below: representative real-time bioluminescence assays from zebrafish PAC-2 cells transfected with the *E2F/D-box*_*ddb2*_*-Luc* and *D-box*_*ddb2*_*-Luc* reporter respectively, and treated in DD with 300 μM H_2_O_2_ at the time points indicated by the blue arrows. Bioluminescence (CPS) is plotted on the y-axes and time (hrs) on the x-axes. Each time-point represents the mean of n = 8 ± s.d.. White and black bars below each panel represent the light and dark periods, respectively.
(TIF)

S1 Table Statistical analysis results. (DOCX)

S2 Table Genbank accession numbers for *ddb2* genes in fish species. (DOCX)

S3 Table *ddb2* promoter reporter constructs. (DOCX)

S4 Table Primer sequences for qRT-PCR analysis. (DOCX)

S1 Dataset Data used to make all the figures and statistical analyses. (XLSX)
