# Supplementary figures and images for: Regulation of ddb2 expression in blind cavefish and zebrafish reveals plasticity in the control of sunlight-induced DNA damage repair
Source: PLoS Genet. 2021 Feb 5;17(2):e1009356. doi: 10.1371/journal.pgen.1009356 (PMC7891740; doi:10.1371/journal.pgen.1009356)

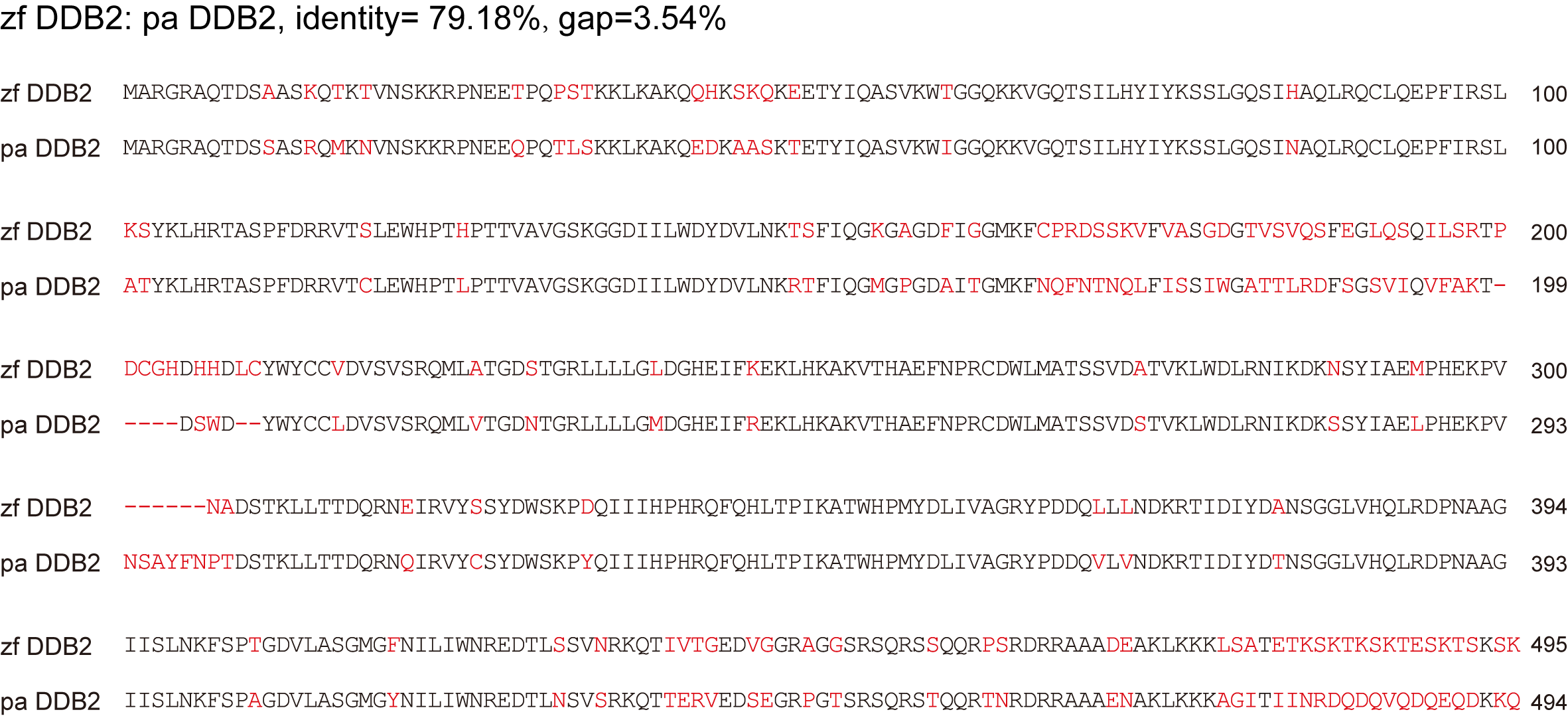

Supplement: S1 Fig — The black fonts denote identical sequences while the red text represent the mismatched amino acids in the sequence alignment. The locations of the aligned sequences are indicated on the right side of each sequence. (The cavefish ddb2 coding sequence reported in this article has been deposited in GenBank under accession number: Genbank MN907102). (TIF) [file pgen.1009356.s001.tif]

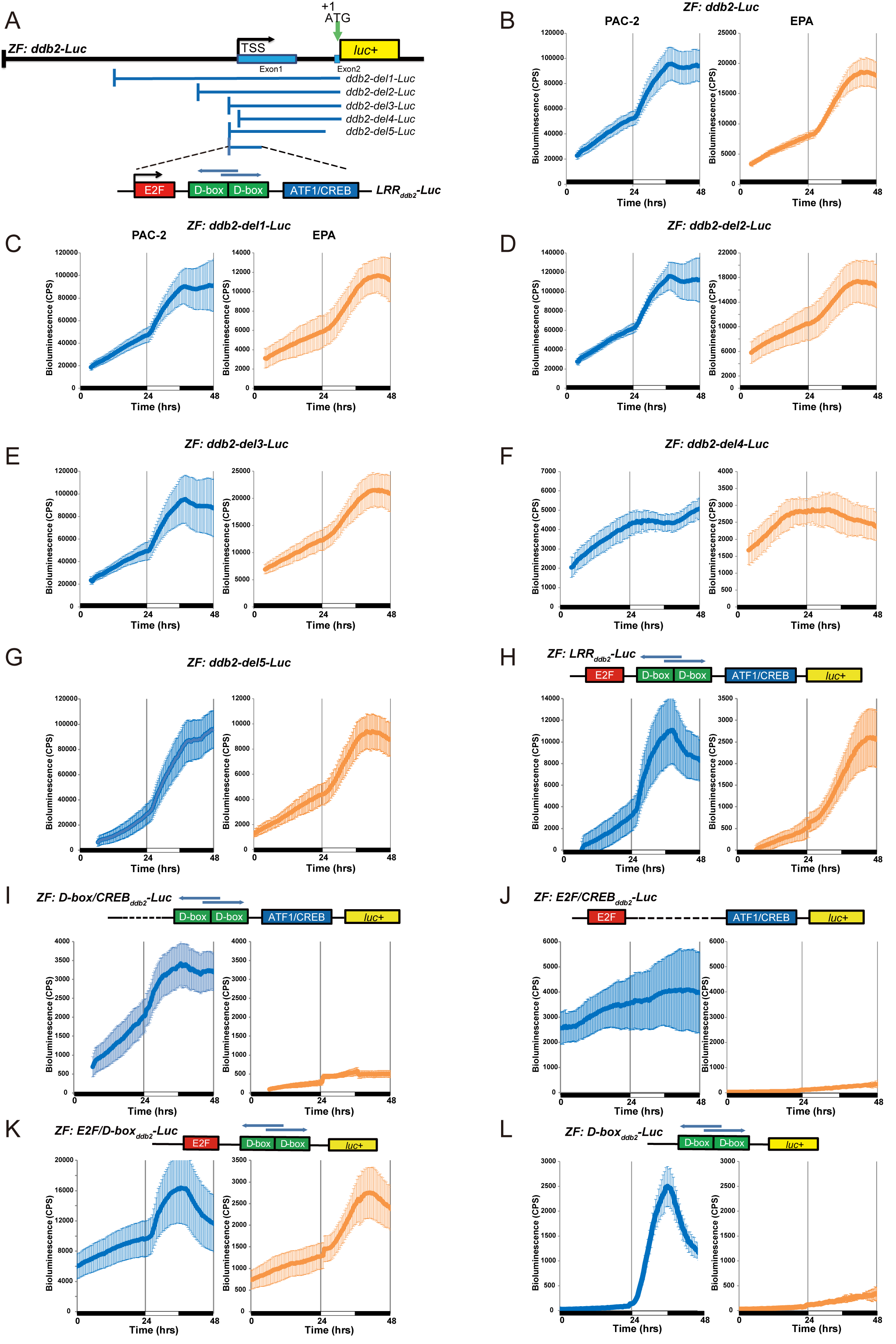

Supplement: S2 Fig — (A) Schematic representation of the various zebrafish ddb2 promoter luciferase reporter constructs analyzed. The position of exon sequences, the transcription start site, ATG translation start codon and the luciferase reporter gene (luc+) are indicated. Below, within the context of the minimal, light-responsive promoter construct LRRddb2-Luc are indicated the E2F site, D-box and ATF1/CREB enhancer elements by red, green and blue rectangles, respectively. Blue arrows above the D-boxes indicate their orientation. (B-G) Real-time bioluminescence assays from zebrafish PAC-2 (blue traces) and cavefish EPA cells (orange traces) transfected with the various zebrafish ddb2 promoter luciferase reporter constructs. Bioluminescence (CPS) is plotted on the y-axes and time (hrs) on the x-axes. Each time-point represents the mean of n = 8 ± s.d.. Black and white bars along the x-axes show dark and light periods. (H-L) Real-time bioluminescence assays from PAC-2 (blue traces) and EPA cells (orange traces) transfected with the sub-deletion constructs derived from LRRddb2-Luc where individual enhancers are deleted. Above each panel is a schematic representation of each construct. Bioluminescence data is presented as described for panels B-G. (TIF) [file pgen.1009356.s002.tif]

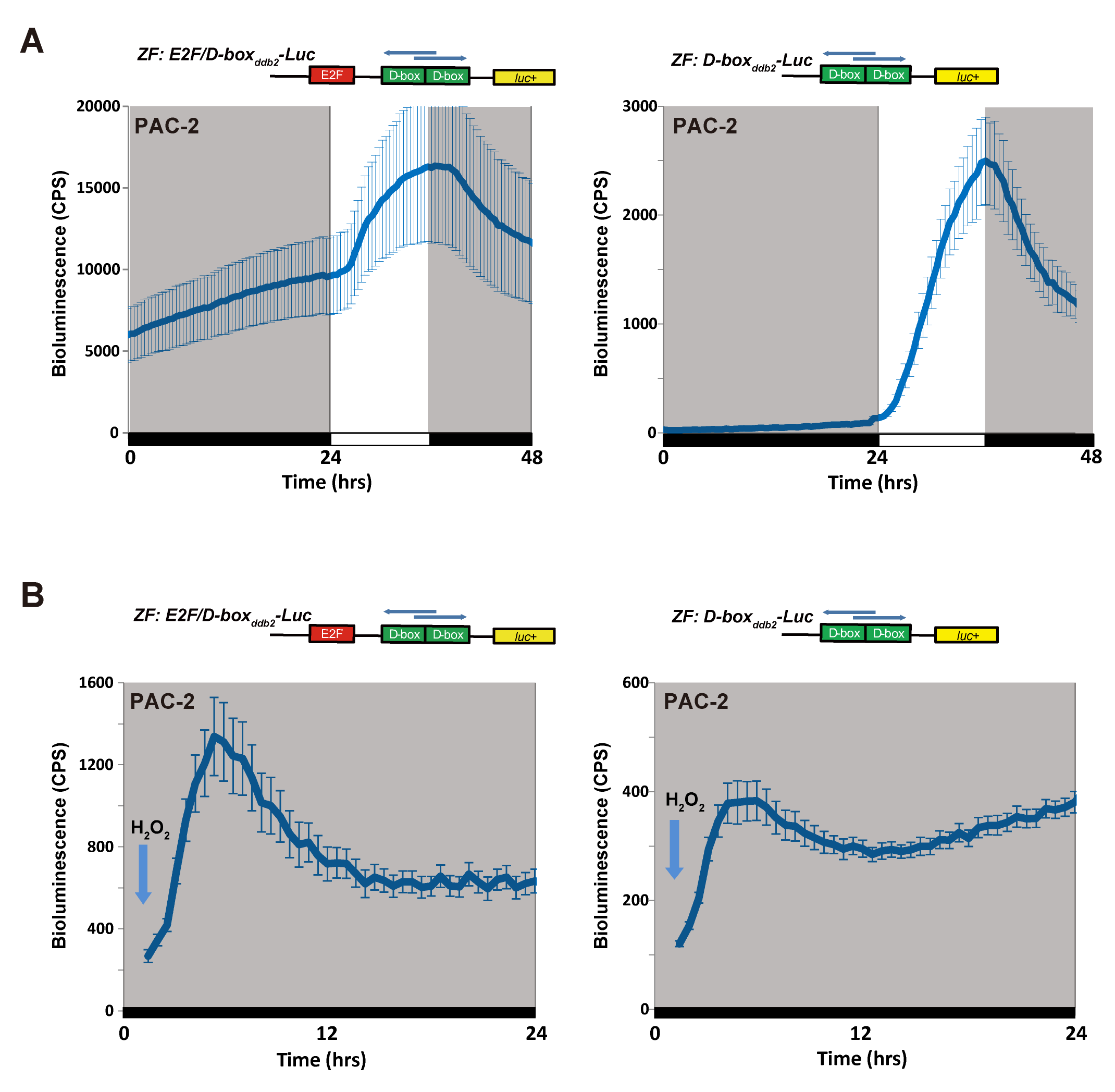

Supplement: S3 Fig — (A,B) Above: Schematic representation of the zebrafish E2F/D-boxddb2-Luc (left panel) and D-boxddb2-Luc (right panel) reporter. (A) Below: representative real-time bioluminescence assays from zebrafish PAC-2 cells transfected with corresponding luciferase reporter vector and exposed to 12 hours of light within a period of constant darkness. (B) Below: representative real-time bioluminescence assays from zebrafish PAC-2 cells transfected with the E2F/D-boxddb2-Luc and D-boxddb2-Luc reporter respectively, and treated in DD with 300 μM H2O2 at the time points indicated by the blue arrows. Bioluminescence (CPS) is plotted on the y-axes and time (hrs) on the x-axes. Each time-point represents the mean of n = 8 ± s.d.. White and black bars below each panel represent the light and dark periods, respectively. (TIF) [file pgen.1009356.s003.tif]
