# Supplementary material for: Regulation of ddb2 expression in blind cavefish and zebrafish reveals plasticity in the control of sunlight-induced DNA damage repair
Source: PLoS Genet. 2021 Feb 5;17(2):e1009356. doi: 10.1371/journal.pgen.1009356 (PMC7891740; doi:10.1371/journal.pgen.1009356)
Supplement: S1 Table — (DOCX) [file pgen.1009356.s004.docx]

**S1 Table. Statistical analysis results**

| Experiment/Figure |  | Test method | Significance | n |
| --- | --- | --- | --- | --- |
| Comet assay: ZF_160 J/m^2^_DD (Fig 1A) |  | Kruskal-Wallis test | ANOVA *P*<0.001 | 300 |
|  | Ctrl vs. 0h | Dunn's multiple comparisons test | *P*<0.001 *** |  |
|  | Ctrl vs. 1h | - | *P*<0.001 *** |  |
|  | Ctrl vs. 2h | - | *P*<0.001 *** |  |
|  | Ctrl vs. 4h | - | *P*<0.001 *** |  |
| Comet assay: PA_160 J/m^2^_DD (Fig 1B) |  | Kruskal-Wallis test | ANOVA *P*<0.001 | 300 |
|  | Ctrl vs. 0h | Dunn's multiple comparisons test | *P*<0.001 *** |  |
|  | Ctrl vs. 1h | - | *P*<0.001 *** |  |
|  | Ctrl vs. 2h | - | *P*<0.001 *** |  |
|  | Ctrl vs. 4h | - | *P*<0.001 *** |  |
| Comet assay: ZF_640 J/m^2^_DD (Fig 1C) |  | Kruskal-Wallis test | ANOVA *P*<0.001 | 300 |
|  | Ctrl vs. 0h | Dunn's multiple comparisons test | *P*<0.001 *** |  |
|  | Ctrl vs. 1h | - | *P*<0.001 *** |  |
|  | Ctrl vs. 2h | - | *P*<0.001 *** |  |
|  | Ctrl vs. 4h | - | *P*<0.001 *** |  |
| Comet assay: PA_640 J/m^2^_DD (Fig 1D) |  | Kruskal-Wallis test | ANOVA *P*<0.001 | 300 |
|  | Ctrl vs. 0h | Dunn's multiple comparisons test | *P*<0.001 *** |  |
|  | Ctrl vs. 1h | - | *P*<0.001 *** |  |
|  | Ctrl vs. 2h | - | *P*<0.001 *** |  |
|  | Ctrl vs. 4h | - | *P*<0.001 *** |  |
| *6-4 photolyase*-visible light induction (Fig 1E) |  | 2 way ANOVA | F=2499; *P*<0.001 | 3 |
|  | ZF DD vs. 0.5h | Sidak's multiple comparisons test | *P*=0.9769 ns |  |
|  | DD vs. 1h | - | *P*<0.001 *** |  |
|  | DD vs. 3h | - | *P*<0.001 *** |  |
|  | DD vs. 6h | - | *P*<0.001 *** |  |
|  | DD vs. 9h | - | *P*<0.001 *** |  |
|  | PA DD vs. 0.5h | Sidak's multiple comparisons test | *P*>0.05 ns |  |
|  | DD vs. 1h | - | *P*>0.05 ns |  |
|  | DD vs. 3h | - | *P*>0.05 ns |  |
|  | DD vs. 6h | - | *P*>0.05 ns |  |
|  | DD vs. 9h | - | *P*>0.05 ns |  |
| *ddb2*-visible light induction (Fig 1F) |  | 2 way ANOVA | F=41.38; *P*<0.001 | 3 |
|  | ZF DD vs. 0.5h | Sidak's multiple comparisons test | *P*=0.999 ns |  |
|  | DD vs. 1h | - | *P*=0.962 ns |  |
|  | DD vs. 3h | - | *P*<0.001 *** |  |
|  | DD vs. 6h | - | *P*<0.001 *** |  |
|  | DD vs. 9h | - | *P*<0.001 *** |  |
|  | PA DD vs. 0.5h | Sidak's multiple comparisons test | *P*=0.964 ns |  |
|  | DD vs. 1h | - | *P*=0.871 ns |  |
|  | DD vs. 3h | - | *P*<0.001 *** |  |
|  | DD vs. 6h | - | *P*<0.001 *** |  |
|  | DD vs. 9h | - | *P*<0.001 *** |  |
| *ddb2*-ROS induction (Fig 3A) |  | 2 way ANOVA | F=15.19; *P*<0.001 | 3 |
|  | ZF DD vs. 0.5h | Sidak's multiple comparisons test | *P*=0.995 ns |  |
|  | DD vs. 1h | - | *P*>0.05 ns |  |
|  | DD vs. 3h | - | *P*=0.003 ** |  |
|  | DD vs. 6h | - | *P*<0.001 *** |  |
|  | DD vs. 9h | - | *P*<0.001 *** |  |
|  | PA DD vs. 0.5h | Sidak's multiple comparisons test | *P*>0.05 ns |  |
|  | DD vs. 1h | - | *P*>0.05 ns |  |
|  | DD vs. 3h | - | *P*=0.301 ns |  |
|  | DD vs. 6h | - | *P*<0.001 *** |  |
|  | DD vs. 9h | - | *P*<0.001 *** |  |
| *6-4 photolyase*-ROS induction (Fig 3B) |  | 2 way ANOVA | F=69.62; *P*<0.001 | 3 |
|  | ZF DD vs. 0.5h | Sidak's multiple comparisons test | *P*=0.452 ns |  |
|  | DD vs. 1h | - | *P*=0.999 ns |  |
|  | DD vs. 3h | - | *P*=0.085 ns |  |
|  | DD vs. 6h | - | *P*<0.001 *** |  |
|  | DD vs. 9h | - | *P*<0.001 *** |  |
|  | PA DD vs. 0.5h | Sidak's multiple comparisons test | *P>*0.999 ns |  |
|  | DD vs. 1h | - | *P>*0.999 ns |  |
|  | DD vs. 3h | - | *P*=0.999 ns |  |
|  | DD vs. 6h | - | *P*=0.963 ns |  |
|  | DD vs. 9h | - | *P*>0.999 ns |  |
| NAC effects on light induction of *ddb2* (Fig 3C) | Student’s t-test (unpaired, two tailed) | |  | 3 |
|  | Ctrl vs. NAC | ZFPAC-2 3h | *P*<0.001 *** |  |
|  | Ctrl vs. NAC | ZF PAC-2 6h | *P*<0.001 *** |  |
|  | Ctrl vs. NAC | PA EPA 3h | *P*=0.022 * |  |
|  | Ctrl vs. NAC | PA EPA 6h | *P*<0.001 *** |  |
| *ddb2*-UV induction (Fig 4A) |  | 2 way ANOVA | F=53.54; *P*<0.001 | 3 |
|  | ZF Ctrl vs. 3h-12h | Sidak's multiple comparisons test | *P*>0.05 ns |  |
|  | Ctrl vs. 24h-60h | - | *P*<0.001 *** |  |
|  | PA Ctrl vs. 3h | Sidak's multiple comparisons test | *P*=0.540 ns |  |
|  | Ctrl vs. 6h | - | *P*=0.049 * |  |
|  | Ctrl vs. 9h-60h | - | *P*<0.001 *** |  |
| *6-4 photolyase*-UV induction (Fig 4B) |  | 2 way ANOVA | F=519.8; *P*<0.001 | 3 |
|  | ZF Ctrl vs. 3h | Sidak's multiple comparisons test | *P*=0.761 ns |  |
|  | Ctrl vs. 6h | - | *P*=0.004 ** |  |
|  | Ctrl vs. 9h-60h | - | *P*<0.001 *** |  |
|  | PA Ctrl vs. 3h-6h | - | *P*>0.05 ns |  |
|  | Ctrl vs. 9h | - | *P*=0.047 * |  |
|  | Ctrl vs. 12h | - | *P*=0.001 ** |  |
|  | Ctrl vs. 24h-36h | - | *P*<0.001 *** |  |
|  | Ctrl vs. 48h | - | *P*=0.214 ns |  |
|  | Ctrl vs. 54h | - | *P*=0.016 * |  |
|  | Ctrl vs. 60h | - | *P*=0.015 * |  |
| NAC effects on UV induction of *ddb2* (Fig 4C) | Student’s t-test (unpaired, two tailed) | |  | 3 |
|  | Ctrl vs. NAC | ZF PAC-2 24h | *P*<0.001 *** |  |
|  | Ctrl vs. NAC | ZF PAC-2 36h | *P*<0.001 *** |  |
|  | Ctrl vs. NAC | PA EPA 24h | *P*<0.001 *** |  |
|  | Ctrl vs. NAC | PA EPA 36h | *P*<0.001 *** |  |
| mRNA stability of ZF *CPD photolyase* (Fig 5A) |  | 2 way ANOVA | F=14.12; *P*<0.001 | 3 |
|  | Ctrl vs. UV-C 2h | Sidak's multiple comparisons test | *P*<0.001 *** |  |
|  | Ctrl vs. UV-C 4h | - | *P*<0.001 *** |  |
|  | Ctrl vs. UV-C 6h | - | *P*<0.001 *** |  |
|  | Ctrl vs. UV-C 8h | - | *P*<0.001 *** |  |
| mRNA stability of PA *CPD photolyase* (Fig 5B) |  | 2 way ANOVA | F=1.102; *P*=0.38 | 3 |
|  | Ctrl vs. UV-C 2h | Sidak's multiple comparisons test | *P*>0.99 ns |  |
|  | Ctrl vs. UV-C 4h | - | *P*=0.47 ns |  |
|  | Ctrl vs. UV-C 6h | - | *P*=0.81 ns |  |
|  | Ctrl vs. UV-C 8h | - | *P*=0.96 ns |  |
| mRNA stability of ZF *6-4 photolyase* (Fig 5C) |  | 2 way ANOVA | F=66.09; *P*<0.001 | 3 |
|  | Ctrl vs. UV-C 2h | Sidak's multiple comparisons test | *P*<0.001 *** |  |
|  | Ctrl vs. UV-C 4h | - | *P*<0.001 *** |  |
|  | Ctrl vs. UV-C 6h | - | *P*<0.001 *** |  |
|  | Ctrl vs. UV-C 8h | - | *P*<0.001 *** |  |
| mRNA stability of PA *6-4 photolyase* (Fig 5D) |  | 2 way ANOVA | F=1.051; *P*=0.41 | 3 |
|  | Ctrl vs. UV-C 2h | Sidak's multiple comparisons test | *P*>0.05 ns |  |
|  | Ctrl vs. UV-C 4h | - | *P*=0.93 ns |  |
|  | Ctrl vs. UV-C 6h | - | *P*=0.54 ns |  |
|  | Ctrl vs. UV-C 8h | - | *P*=0.62 ns |  |
| mRNA stability of ZF *ddb2* (Fig 5E) |  | 2 way ANOVA | F=28.71; *P*<0.001 | 3 |
|  | Ctrl vs. UV-C 2h | Sidak's multiple comparisons test | *P*<0.001 *** |  |
|  | Ctrl vs. UV-C 4h | - | *P*<0.001 *** |  |
|  | Ctrl vs. UV-C 6h | - | *P*<0.001 *** |  |
|  | Ctrl vs. UV-C 8h | - | *P*<0.001 *** |  |
| mRNA stability of PA *ddb2* (Fig 5F) |  | 2 way ANOVA | F=18.35; *P*<0.001 | 3 |
|  | Ctrl vs. UV-C 2h | Sidak's multiple comparisons test | *P*<0.001 *** |  |
|  | Ctrl vs. UV-C 4h | - | *P*<0.001 *** |  |
|  | Ctrl vs. UV-C 6h | - | *P*=0.001 ** |  |
|  | Ctrl vs. UV-C 8h | - | *P*=0.011 * |  |
| mRNA stability of ZF *xpc* (Fig 5G) |  | 2 way ANOVA | F=13.19; *P*<0.001 | 3 |
|  | Ctrl vs. UV-C 2h | Sidak's multiple comparisons test | *P*<0.001 *** |  |
|  | Ctrl vs. UV-C 4h | - | *P*<0.001 *** |  |
|  | Ctrl vs. UV-C 6h | - | *P*<0.001 *** |  |
|  | Ctrl vs. UV-C 8h | - | *P*<0.001 *** |  |
| mRNA stability of PA *xpc* (Fig 5H) |  | 2 way ANOVA | F=7.09; *P*=0.001 | 3 |
|  | Ctrl vs. UV-C 2h | Sidak's multiple comparisons test | *P*=0.69 ns |  |
|  | Ctrl vs. UV-C 4h | - | *P*<0.001 *** |  |
|  | Ctrl vs. UV-C 6h | - | *P*<0.001 *** |  |
|  | Ctrl vs. UV-C 8h | - | *P*=0.09 ns |  |
