# Supplementary material for: Regulation of ddb2 expression in blind cavefish and zebrafish reveals plasticity in the control of sunlight-induced DNA damage repair
Source: PLoS Genet. 2021 Feb 5;17(2):e1009356. doi: 10.1371/journal.pgen.1009356 (PMC7891740; doi:10.1371/journal.pgen.1009356)
Supplement: S2 Table — (DOCX) [file pgen.1009356.s005.docx]

**S2 Table. Genbank assession numbers for *ddb2* *gene*s in fish species.**

| Species | Ensembl No. |
| --- | --- |
| Zebrafish (*Danio rerio*) | ENSDARG00000041140 |
| Amazon molly (*Poecilia formosa*) | ENSPFOG00000010390 |
| Japanese Medaka (*Oryzias latipes*) | ENSORLG00020008297 |
| Chinese Medaka (*Oryzias sinensis*) | ENSOSIG00000010596 |
| Cod (*Gadus morhua*) | ENSGMOG00000017517 |
| Guppy (*Poecilia reticulata*) | ENSPREG00000011139 |
| Fugu (*Takifugu rubripes*) | ENSTRUG00000015270 |
| Common carp (*Cyprinus carpio*) | ENSCCRG00000018879 |
| Stickleback (*Gasterosteus aculeatus*) | ENSGACG00000005252 |
| Platyfish (*Xiphophorus maculatus*) | ENSXMAG00000003694 |
| Mexican tetra (*Astyanax mexicanus*) | ENSAMXG00000000525 |
| Pachon cavefish (*Astyanax mexicanus*) | ENSAMXG00005016420 |
| Gold fish (*Carassius auratus*) | ENSCARG00000016846 |
