# Supplementary material for: Regulation of ddb2 expression in blind cavefish and zebrafish reveals plasticity in the control of sunlight-induced DNA damage repair
Source: PLoS Genet. 2021 Feb 5;17(2):e1009356. doi: 10.1371/journal.pgen.1009356 (PMC7891740; doi:10.1371/journal.pgen.1009356)
Supplement: S3 Table — (DOCX) [file pgen.1009356.s006.docx]

**S3 Table. *ddb2* promoter reporter constructs.**

| Construct name | Position and length | Primers used for construction |
| --- | --- | --- |
| *ddb2-Luc* | -862bp to +18bp (880bp) | F: AGTTTAAAGAAAGTAGCCAAAATCA  R: CTGTCTGAGCTCTTCCTCGC |
| *ddb2-del1-Luc* | -585bp to +18bp (603bp) | F: CCAAAACAACTGTACTGCTG  R: GAGCTCGGTACCTATCGATAG |
| *ddb2-del2-Luc* | -434bp to +18bp (452bp) | F: CTTATGTAGCTGTACAATGAATTTG  R: GAGCTCGGTACCTATCGATAG |
| *ddb2-del3-Luc* | -293bp to +18bp (311bp) | F: TCTAGTGGCACCTAGTGG  R: GAGCTCGGTACCTATCGATA |
| *ddb2-del4-Luc* | -231bp to +18bp (249bp) | F: GTTTATCGCACTCAACGATTAC  R: GAGCTCGGTACCTATCGA |
| *ddb2-del5-Luc* | -271bp to -97bp (197bp) | F: GAGCTCTTACGCGTGCTA  R: TCATATAGCAATTGCATTACTCG |
| *LRR_ddb2_-Luc* | -271bp to -223bp (49bp) | F: GAGCTCTTACGCGTGCTA  R: TACATTCAGTGACGTAGAAGATTG |
| *D-box/CREB_ddb2_-Luc* | *LRR_ddb2_-Luc* E2F binding site (ATTGTTTATCGCA) deleted | F: CTCAACGATTACAAAACATTCAATC  R: TTATCCACTAGGTGCCAC |
| *E2F/CREB_ddb2_-Luc* | *LRR_ddb2_-Luc* overlapping 2×D-box (TTACAAAACATT) deleted | F: AATCTTCTACGTCACTGAATG  R: TCGTTGAGTGCGATAAAC |
| *E2F/D-box_ddb2_-Luc* | *LRR_ddb2_-Luc* ATF1/CREB site (ACGTCAC) deleted | F: CTGAATGTAGTGAAAATTCG  R: AGAAGATTGAATGTTTTGTAATC |
| *D-box_ddb2_-Luc* | *D-box/CREB_ddb2_-Luc* ATF1/CREB site (ACGTCAC) deleted | F: CTGAATGTAGTGAAAATTCG  R: AGAAGATTGAATGTTTTGTAATC |
