# Supplementary material for: Regulation of ddb2 expression in blind cavefish and zebrafish reveals plasticity in the control of sunlight-induced DNA damage repair
Source: PLoS Genet. 2021 Feb 5;17(2):e1009356. doi: 10.1371/journal.pgen.1009356 (PMC7891740; doi:10.1371/journal.pgen.1009356)
Supplement: S4 Table — (DOCX) [file pgen.1009356.s007.docx]

**S4 Table. Primer sequences for qRT-PCR analysis.**

| **Gene** | **zebrafish (*Danio rerio*)** | **cavefish (*Phreatichthys andruzzii*)** |
| --- | --- | --- |
| ***β-actin*** | F: GATGAGGAAATCGCTGCCCT  R. GTCCTTCTGTCCCATGCCAA | F: GATGAGGAAATCGCTGCCCT  R. GTCCTTCTGTCCCATGCCAA |
| ***6-4 photolyase*** | F: AATGGCAAGACTCCCATGAC  R: GTGGCCCTAAGGATGACGTA | F: CTGCAGAGGTCCTTCCAAAG  R: GCTTTCCGTTGTTCTCTTCG |
|  | F: GCAAAGCAGAGCTCACGTAAT  R: AGGAACAGGGGGTAGATGTGT |  |
|  | F: CAAGGGGCTGCGTTTGCATGAT  R: GAACAGGGGGTAGATGTGTCT | F: CAAGGGGCTGCGTTTGCATGAT  R: GAACAGGGGGTAGATGTGTCT |
| ***ddb2*** | F: TCGGTCTTGCTCTTGGTCTT  R: GAGGCAGAGCTGGAGGTTC | F: AGGGCTCAGACAGATTCCTCT  R: CCTCCAATCCATTTGACACTTGC |
|  | F: ACAGACTCTGCTGCATCGAA  R: AGAATGCTTGTTTGGCCCAC | F: ACAGACTCTGCTGCATCGAA  R: AGAATGCTTGTTTGGCCCAC |
| ***CPD photolyase*** | F: GAGTTCAGGGCATCACGTC  R: GCACTGATCGTCGACTTCA | F: GCTGCTGAAGGAAGTGACC  R: CAGTAGAGGACCCCATCAG |
|  | F: AGGGATGCGAGTTCAACAA  R: TTCTCAGCCAAAGCCAGC | F: AGGGATGCGAGTTCAACAA  R: TTCTCAGCCAAAGCCAGC |
|  | F: GCTGGCTTTGGCTGAGAA  R: ACATTATGTGCATCAACCTG | F: GCTGGCTTTGGCTGAGAA  R: ACATTATGTGCATCAACCTG |
|  | F: GCATGTACTGGGCAAAGA  R: AGACAAACGGTCATTCAGAT | F: GCATGTACTGGGCAAAGA  R: AGACAAACGGTCATTCAGAT |
| ***xpc*** | F: GCCAACATCCGTCTCAGAAT  R: GAACGGTTGGAAAAACCAAG | F: GTGGACTCGACTGAACTAGC  R: CAGGTCAGACTCACAGCAC |
